# Human West Nile Virus Lineage 2 Infection: Epidemiological, Clinical, and Virological Findings

**DOI:** 10.3390/v12040458

**Published:** 2020-04-18

**Authors:** Monia Pacenti, Alessandro Sinigaglia, Elisa Franchin, Silvana Pagni, Enrico Lavezzo, Fabrizio Montarsi, Gioia Capelli, Luisa Barzon

**Affiliations:** 1 Microbiology and Virology Unit, Padova University Hospital, I-35128 Padova, Italy; monia.pacenti@aopd.veneto.it (M.P.); elisa.franchin@unipd.it (E.F.); silvana.pagni@unipd.it (S.P.); 2 Department of Molecular Medicine, University of Padova, I-35121 Padova, Italy; alessandro.sinigaglia@unipd.it (A.S.); enrico.lavezzo@unipd.it (E.L.); 3 Istituto Zooprofilattico Sperimentale delle Venezie, I-35020 Legnaro PD, Italy; fmontarsi@izsvenezie.it (F.M.); gcapelli@izsvenezie.it (G.C.)

**Keywords:** West Nile virus, encephalitis, fever, diagnosis, symptoms, outbreak, surveillance, mosquitoes, epidemiology, neuroinvasive disease

## Abstract

West Nile virus (WNV) lineage 2 is expanding and causing large outbreaks in Europe. In this study, we analyzed the epidemiological, clinical, and virological features of WNV lineage 2 infection during the large outbreak that occurred in northern Italy in 2018. The study population included 86 patients with neuroinvasive disease (WNND), 307 with fever (WNF), and 34 blood donors. Phylogenetic analysis of WNV full genome sequences from patients’ samples showed that the virus belonged to the widespread central/southern European clade of WNV lineage 2 and was circulating in the area at least since 2014. The incidence of WNND and WNF progressively increased with age and was higher in males than in females. Among WNND patients, the case fatality rate was 22%. About 70% of blood donors reported symptoms during follow-up. Within the first week after symptom onset, WNV RNA was detectable in the blood or urine of 80% of patients, while 20% and 40% of WNND and WNF patients, respectively, were WNV IgM-seronegative. In CSF samples of WNND patients, WNV RNA was typically detectable when WNV IgM antibodies were absent. Blunted or no WNV IgM response and high WNV IgG levels were observed in seven patients with previous flavivirus immunity.

## 1. Introduction

West Nile virus (WNV) is a mosquito-borne flavivirus transmitted by *Culex* mosquitoes among a variety of bird species [1]. Humans are dead-end hosts, incidentally infected through a mosquito bite. Infection in humans is generally asymptomatic; 20–30% of cases develop influenza-like illness, defined as West Nile fever (WNF), while less than 1% of infected individuals, mainly elderly and immunocompromised patients, develop neuroinvasive disease (WNND), i.e., encephalitis, meningitis, or acute flaccid paralysis. In patients with WNND, mortality is about 10% and severe sequelae persist in 20–40% of those who survive [2].

Nine different evolutionary lineages of WNV have been described so far, but only lineages 1 and 2 have been associated with disease in humans [3]. In Europe, the most widespread WNV strains belong to the central/southern European clade of WNV lineage 2, which emerged in central Europe in 2004 [4]. Then, the virus dispersed from Hungary to western and southern European countries, where it has caused large human outbreaks, such as in Greece, since 2010 [5], and in Italy, since 2013 [6].

In Italy, the first human cases of WNV infection, caused by WNV lineage 1, were detected in northern Italy in 2008 [7]. Since then, outbreaks of WNV infection have occurred every year in northern Italy [7]. Until 2011, only WNV lineage 1 strains have been detected in Italy by human, veterinary, and entomological surveillance and involved in human outbreaks. In Italy, the first incursions of WNV lineage 2, the central/southern European clade, were observed in 2011, when sporadic infections in humans and birds were reported [7]. The first large human outbreaks of WNV lineage 2 infection occurred in 2013 in northern Italy, where the virus still co-circulated with WNV lineage 1 in some areas [6]. During the following years, WNV lineage 2 progressively expanded its geographic range and replaced WNV lineage 1 [8]. Notably, in 2014, eastern European WNV lineage 2 related to the Volgograd 2007 strain was also detected in mosquito pools in northern Italy [9].

Usutu virus (USUV), a mosquito-borne flavivirus genetically related to WNV, is also endemic in Italy. The first evidence of its activity in the country dates back to 1996, when it caused massive mortality among blackbirds [10]. WNV and USUV share the same transmission cycle and co-circulate in the same areas in Italy [11,12], but USUV is considered less pathogenic for humans than WNV [12].

The Italian Ministry of Health publishes an annually revised surveillance plan, which aims to reduce the risk of WNV transmission to humans by detecting viral circulation early and triggering both vector-control measures and safety measures for substances of human origin [13]. In the Veneto region, northeastern Italy, where WNV is endemic and has caused human outbreaks since 2008 [14], the surveillance plan includes passive surveillance of human cases of WNV fever, besides surveillance of WNND, as well as entomological monitoring [15]. Since 2017, the national and regional surveillance plans include the surveillance of USUV. Human cases of USUV infection identified in the Veneto region in 2018 and the results of USUV entomological surveillance have already been described [12]. Based on these plans, all suspected autochthonous human cases of arbovirus infection, all organ, stem cell, tissue, and blood donors with a positive WNV NAT screening test, were investigated at the regional reference laboratory of the Veneto Region (Microbiology and Virology Unit, Padova University Hospital) for laboratory confirmation.

In 2018, probably as a consequence of an earlier onset of the transmission season due to warm temperatures during spring, large outbreaks of WNV infection and expansion of the geographic range of the virus were reported in several European countries, including Italy, Greece, Romania, and Hungary [16,17,18]. Notably, over 2000 human WNV infections were reported in 2018 in European countries, which exceeded the total number of cases reported during the previous seven years [19]. Northern Italy was the most affected area, with 606 human cases of WNV infection reported, including 239 with neuroinvasive disease [20]. Preliminary data on the outbreak have been published by the Italian Institute of Health, highlighting the exceptionally high number of human WNV infections occurring in Italy from June to July 2018 [18].

In this study, we described the epidemiological, clinical, and virological findings in a series of 427 autochthonous confirmed cases of WNV infection from northern Italy, who were investigated at the regional reference laboratory of the Veneto Region in 2018. The aims of this study were to define the characteristics of WNV infection in humans, including demographic aspects, clinical presentation of the disease, the kinetics of WNV RNA in body fluids, the dynamics of the antibody response, and to characterize the WNV lineage 2 strain involved in the outbreak.

## 2. Materials and Methods

### 2.1. Sample and Data Collection

Case definition of WNV infection was according to the national surveillance plan [13]. Confirmed WNV cases were defined as individuals presenting with at least one of the following laboratory criteria: Virus isolation from serum, urine, and/or cerebrospinal fluid (CSF); detection of viral RNA in blood, urine, and/or CSF; detection of a specific IgM antibody response in CSF; high IgM antibody titer; and detection of IgG antibodies in serum and confirmation by neutralization assays. A probable WNV case was defined as an individual with only IgM antibodies detected in serum.

According to the regional surveillance plan, collection of the following biological samples was recommended for laboratory diagnosis of WNV infection: Whole blood and urine for WNV RNA detection within 4 weeks since symptom onset; serum at baseline and after 2 to 3 weeks for WNV IgM and IgG detection; and CSF from patients with neurological symptoms for the detection of WNV RNA and WNV IgM antibodies. In this study, the different sample types were not available from all patients, while additional follow-up samples were collected from several confirmed WNV cases.

The data analyzed in this study included all the laboratory results of confirmed autochthonous WNV cases (86 with WNND, 307 with West Nile fever (WNF), and 34 blood donors) that were obtained at the regional reference laboratory. Information on symptoms and clinical diagnosis were retrieved from case report forms.

In relation to blood donors, we included in this study the 34 donors with positive WNV NAT results who were confirmed with further laboratory testing, according to the case definition. All the 34 blood donors, either with symptomatic or asymptomatic WNV infection, were submitted to a follow-up evaluation, including baseline WNV RNA testing in blood and urine and IgM and IgG antibody detection in serum within 3 days after the index donation, and weekly evaluations during the first month and at month 6 after the index donation.

Suspected and probable cases of WNV infection were not included in this study.

### 2.2. Entomological Surveillance

Entomological surveillance was carried out in the Veneto Region from May to October using 55 CDC traps baited with CO_2_ (IMT^®^ – Italian Mosquito Trap) located all over the region, excluding Belluno Province. The traps were run for one night every two weeks.

### 2.3. Laboratory Methods

Laboratory investigation of suspected WNV infections was carried out as previously described [21]. Briefly, for viral RNA detection, total nucleic acids were purified from 200 μL of whole blood by using a MagNA Pure 96 System (Roche Applied Sciences, Basel, Switzerland) and from 1000 μL of plasma, urine, or cerebrospinal fluid (CSF) by using a NucliSens EasyMag System (BioMerieux, Marcy-l’Étoile, France). Target viral RNA sequences were amplified by in-house real-time RT-PCR methods, which allowed discrimination between WNV lineage 1 and WNV lineage 2, as described [21]. Real-time RT-PCR assays were carried out using the one-step real-time kit (Thermo Fisher Scientific, Waltham, MA, USA) and run on ABI 7900HT Sequence Detection Systems (Thermo Fisher Scientific). In addition, in about 50% of WNV RNA-positive blood or urine samples, identification of the WNV lineage was done by broad-range RT-PCR targeting the NS5 region of flaviviruses [22], followed by cycle sequencing. In four samples with a high WNV RNA load, the full viral genome was sequenced, as reported [6].

The presence of WNV IgM and IgG antibodies in serum and CSF was determined by a commercial ELISA (WNV IgM capture DxSelect e IgG DxSelect, Focus Diagnostics, CA, USA). Serum samples with positive results were further tested for confirmation by the plaque reduction neutralization test (PRNT) against WNV and the microneutralization titer assay against USUV [12].

For entomological surveillance, mosquitoes were morphologically identified, pooled (100 specimens maximum), and screened for flaviviruses by using an in-house developed one-step SYBR green-based real-time RT-PCR assay, as described [9]. All flavivirus RNA-positive mosquito pools were directly sequenced to differentiate WNV, USUV, or other flaviviruses.

### 2.4. Statistical Analysis

Data were presented as mean value ± standard deviation or 95% confidence interval and as a median and interquartile range (IQR). Statistical analyses were conducted using unpaired Student’s t-test, factorial ANOVA, and χ^2^ test. The statistical significance was defined as *p* < 0.05. All statistical analyses were performed by using the Statistica™ software, version 13.4.0.14 (TIBCO Software Inc., Paolo Alto, CA).

### 2.5. Ethics Statement

The cases reported in this study were investigated with routine procedures according to the national surveillance plan for WNV and USUV infection. Therefore, no approval was required from the ethics committee.

## 3. Results

### 3.1. Demographic and Epidemiological Analyses of Human Cases of WNV Infection

In the Veneto region, northern Italy, during the surveillance period (1 June –30 November 2018), of the 1967 cases of suspected autochthonous arbovirus infection, 427 were classified as confirmed WNV infections based on the laboratory test results. These confirmed cases included 86 patients with WNND, 307 with WNF, and 34 blood donors (Table 1). Nineteen patients with WNND died, accounting for 22% of the case fatality rate. The mean age of patients with WNND was significantly higher than that of patients with WNF and the prevalence of WNV disease was significantly higher in males than in females (Table 1).

**Figure 1:**
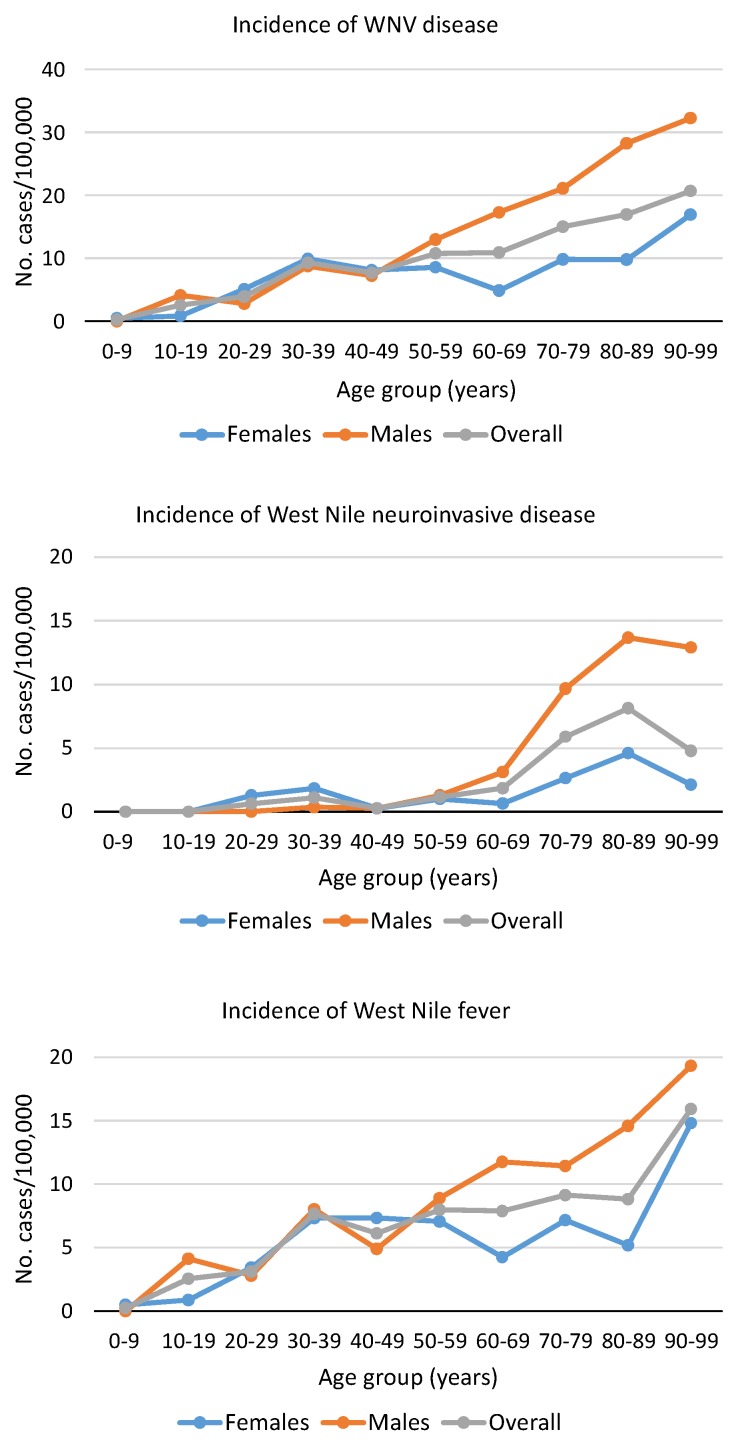
Incidence of WNV disease (i.e., West Nile neuroinvasive disease and West Nile fever), incidence of West Nile neuroinvasive disease, and incidence of West Nile fever according to age and sex, Veneto region, northern Italy, 2018. Data on the population resident in the Veneto region were obtained from ISTAT (Italian Institute of Statistics, www.istat.it).

Normalization of the number of WNV cases against demographic data of the population in the Veneto region showed that the incidence of WNF progressively increased with age, while WNND incidence sharply peaked in subjects older than 70 years of age (Figure 1). Data also showed that the incidence of WNND and WNF was higher in males than in females among patients aged >60 years (Figure 1).

**Figure 2:**
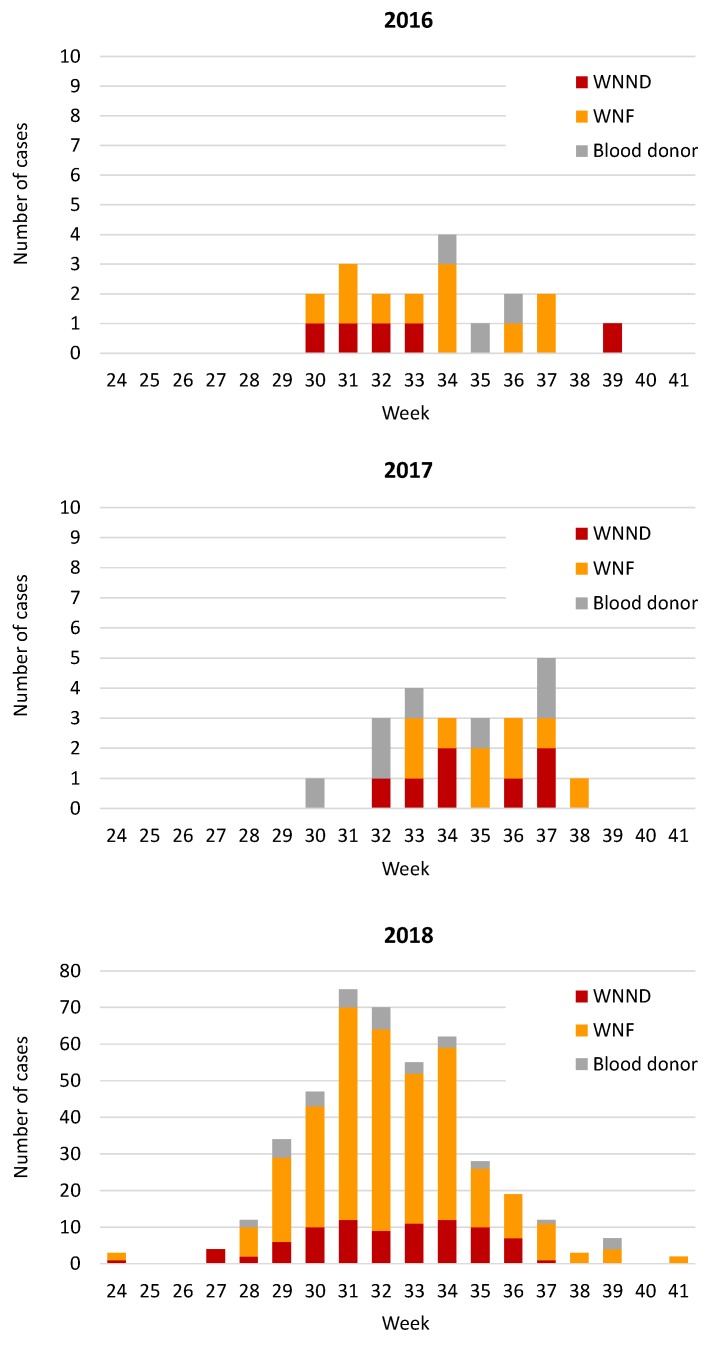
Epidemic curves of human cases of WNV infection according to the week of the onset of symptoms or WNV nucleic acid testing (NAT)-positive index blood donation, Veneto region, northern Italy, 2016, 2017, 2018. Please note the different scales of the Y-axis among graphs. WNND: West Nile neuroinvasive disease; WNF: West Nile fever; blood donor: WNV NAT-positive blood donor.

In 2018, there was an anticipation and a longer duration of the WNV transmission period than in previous years (Figure 2). In particular, the first human case of WNV infection developed symptoms of encephalitis in mid-June and the last case developed symptoms in mid-October. The largest number of cases of infection occurred between mid-July and mid-August, with a peak during the last week of July.

The total number of West Nile cases identified in the Veneto region in 2018 was 20-fold higher than the average number of cases identified in the previous two years (Figure 2). However, considering the incidence of WNV infection in blood donors, which was not affected by variables related to the intensity of surveillance, the increase of WNV cases in 2018 compared to 2016–2017 was 7-fold. The intense training and information to physicians and the population on WNV infection and control measures during the 2018 summer season probably contributed to the increased identification of WNV infections.

**Figure 3:**
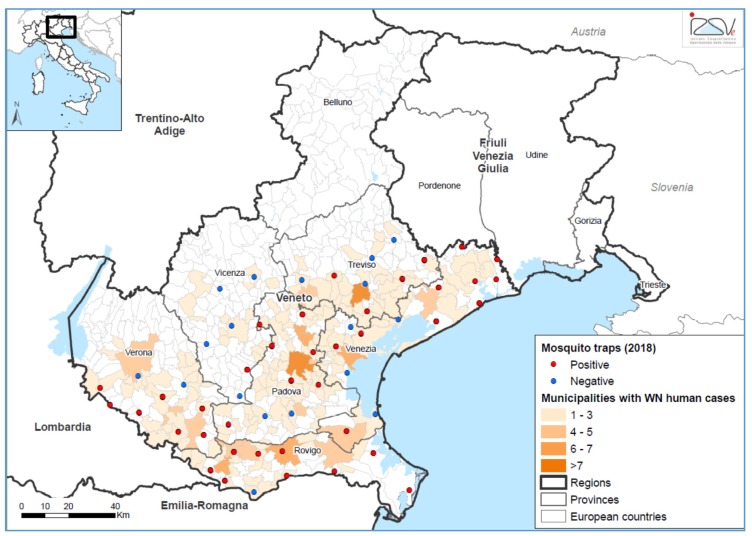
Geographical distribution of West Nile virus (WNV)-positive *Culex* mosquito pools and human cases of WNV infection, Veneto region, northern Italy, 2018. Sites of mosquito traps are represented with dots. Red dots indicate sites where WNV-positive mosquito pools were collected, blue dots indicate sites where no WNV-positive mosquitoes were detected. Colored areas represent municipalities where human cases of WNV infection were present.

Cases were identified in all provinces of the region except Belluno Province, characterized by a mountain territory (Figure 3). The overall incidence of WNND and WNF in the Veneto region was 1.75 and 6.26 per 100,000 population, respectively. The highest incidence was recorded in Rovigo Province, a wetland area near the Po River, with 11.6 cases of WNND and 35 cases of WNF per 100,000 population. Among blood donors, the incidence of WNV infection was 28.9 per 100,000 in the Veneto region and 52.1 per 100,000 in the Rovigo Province.

### 3.2. Entomological Surveillance

Similar to the human surveillance, entomological monitoring also gave the highest rate of WNV-positive mosquito pools in 2018 (*n* = 155; 13%), compared to the past (e.g., 1.8% in 2017). The first *Culex pipiens* mosquito pool was found infected on June 7, one week before the first human case, and the last on September 18, three weeks ahead of the last human case. Of the 55 mosquito trapping sites over the Veneto region, 38 (69%) were found to be positive at least once (Figure 3), confirming the impressive and unusual viral circulation of 2018. In agreement with the human incidence rates, Rovigo Province had the highest rate of positive mosquito pools (15.8%). In all WNV RNA-positive mosquito pools, WNV lineage 2 central/southern European clade was identified, while WNV lineage 1 was not detected.

### 3.3. Clinical Findings

The most common physical findings at admission in WNND patients were fever (97% of patients), motor weakness (61%), headache (47%), and maculopapular rash (26%) (Table 1). Among WNND patients, 40% received a clinical diagnosis of aseptic meningitis, 44% encephalitis, 3% acute flaccid paralysis, and 1% Guillain Barré syndrome. Fever was reported by 86% of WNF patients; other common symptoms included asthenia, rash, headache, arthralgia, and myalgia. Notably, 71% of WNV-positive blood donors reported symptoms during follow-up visits (Table 1). Symptoms started 1–10 days after blood donation and included asthenia (41%), myalgia (35%), headache (35%), and rash (24%). Fever was reported by 26% of blood donors.

### 3.4. Virological Findings

#### 3.4.1. WNV Antibody Dynamics

**Figure 4:**
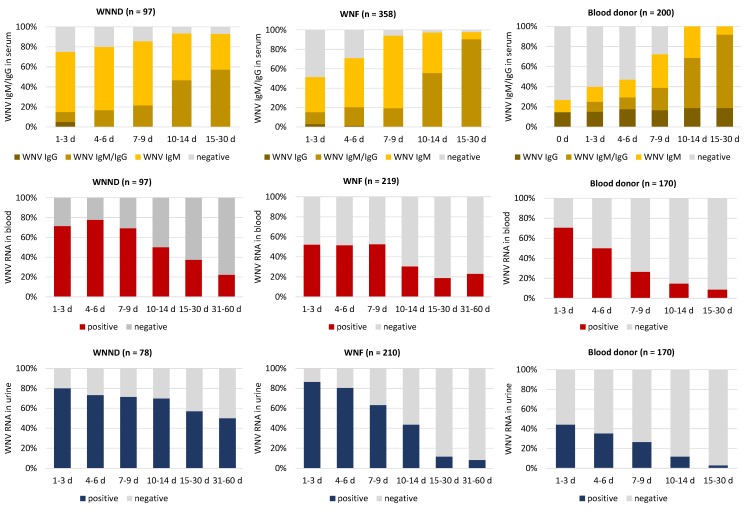
Laboratory findings in human cases of WNV infection according to the time since the onset of symptoms or WNV NAT-positive index blood donation, Veneto region, northern Italy, 2018. Graphs show the percentage of serum samples positive for WNV IgM and/or IgG and percentage of WNV RNA-positive blood and urine samples. The number of tested samples is indicated between brackets. This number is higher than the number of patients because, when available, follow-up samples collected from the same patient were included in the analysis. WNND: West Nile neuroinvasive disease; WNF: West Nile fever.

Antibody dynamics was evaluated by stratification of the WNV IgM and IgG results according to the time since symptom onset (Figure 4). Within the first week after the onset of symptoms, about 20% of patients with WNND and 40% of patients with WNF were seronegative, while WNV IgG antibodies were already detectable in about 20% of both WNF and WNND patients. In all cases confirmed by neutralization assays, the titer of WNV neutralizing antibodies (i.e., the reciprocal of the highest dilution of the serum that reduced by 50% the number of plaques in Vero cells, WNV PRNT50) was ≥40 (generally >80); the titer of USUV neutralizing antibodies (i.e., the reciprocal of the highest dilution of the serum that showed 100% neutralization of the cytopathic effect in the microneutralization assay) was four-fold lower than WNV PRNT50 (generally ≤10). Among WNND cases, no specific IgM and IgG antibody responses were observed in five patients for whom laboratory follow-up data were available. All these patients were immunosuppressed because of underlying malignancies or old age. As expected, the rate of seronegative subjects was higher among WNV NAT-positive blood donors (about 80% at the time of the blood donation and 50% at one week after the donation), since all cases were identified during the acute phase of infection with detectable WNV RNA in blood.

**Figure 5:**
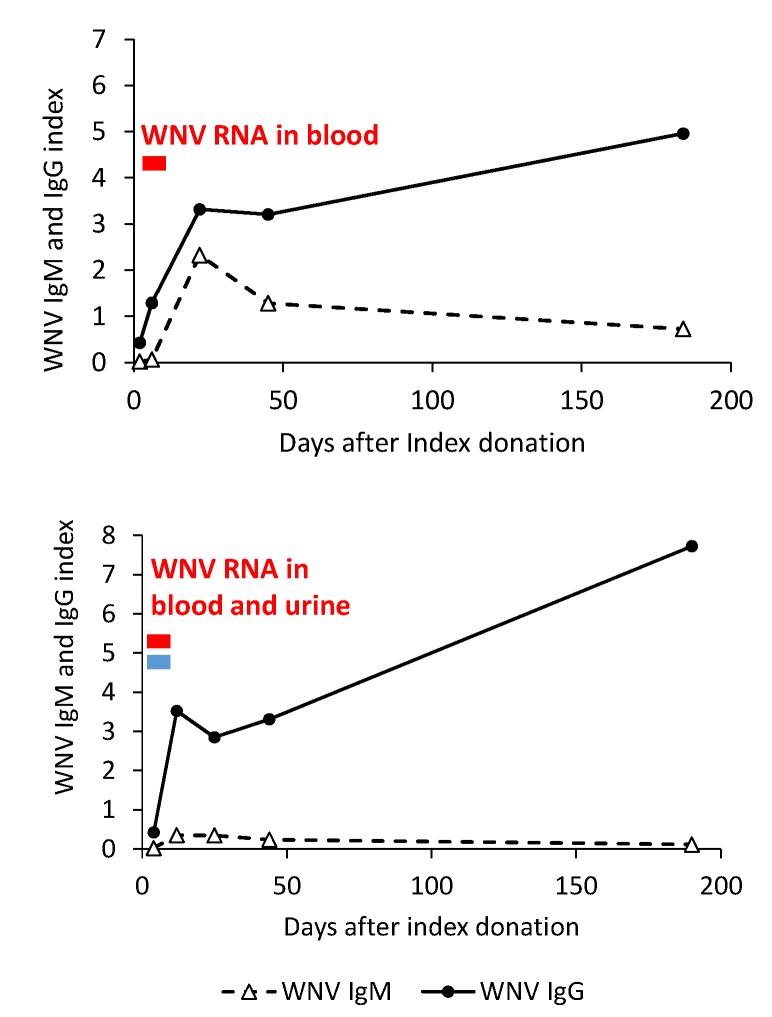
WNV IgM and IgG antibodies in serum and WNV RNA in the blood and urine of two blood donors with WNV infection and a blunted WNV IgM response, Veneto region, northern Italy, 2018.

Notably, seven cases (five blood donors, one cord blood donor, one WNF case) did not develop WNV IgM antibodies during follow-up or had a blunted WNV IgM response but developed WNV IgG antibodies. Five of these cases, who had previous USUV immunity, have already been described [23]. The other two cases were blood donors, including a man in his 60s who developed myalgia and who had been vaccinated against yellow fever 10 years before and a man in his 40s who developed rash, asthenia, and lymphadenopathy and already had high levels (>320) of neutralizing antibodies against WNV and USUV at 12 days after the index donation. Genomic RNA of WNV lineage 2 was detectable in the blood of the first donor and both in the blood and urine in the second donor (Figure 5).

#### 3.4.2. WNV RNA Kinetics in Blood and Urine

**Figure 6:**
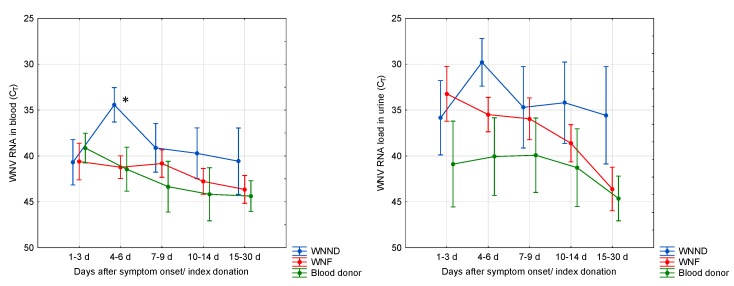
WNV RNA load, reported as the mean threshold cycle (C_T_) values ± 95% CI, in the blood and urine of patients with West Nile neuroinvasive disease (WNND), West Nile fever (WNF), and WNV NAT-positive blood donors (blood donor). The biological samples included in the analyses are the same as those shown in Figure 4. * *p* < 0.05 by factorial ANOVA, WNND vs. WNF and WNND vs. WNV-positive blood donors.

WNV RNA was detectable in the blood of 70–80% of WNND patients and 50% of WNF patients during the first 9 days after symptom onset. WNV RNA persisted in the blood and was still detectable at 30 days after onset in ca 30% of WNND patients and 20% WNF patients (Figure 4). Most WNND and WNF patients had detectable WNV RNA in urine during the first 9 days after onset. WNV RNA persisted in the urine of WNND patients for a longer time than in WNF patients and in blood donors, as it was still detectable at 30 days after onset in over 50% of cases (Figure 4). The levels of WNV RNA in the blood were significantly higher in WNND patients than in WNF patients and in blood donors (Figure 6). Among WNV NAT-positive blood donors, 71% and 44% had detectable WNV RNA in blood and urine, respectively, at the baseline evaluation performed within 3 days after the index donation (Figure 4). The rate of WNV RNA-positive results progressively decreased during follow-up (Figure 4).

#### 3.4.3. WNV RNA and IgM in Cerebrospinal Fluid (CSF)

**Figure 7:**
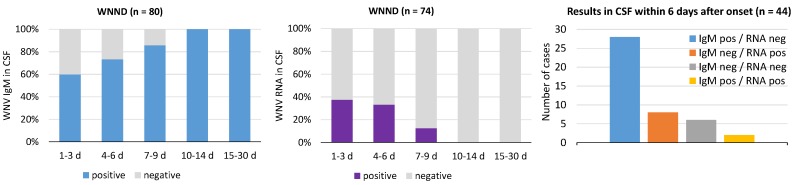
Laboratory findings in cerebrospinal fluid (CSF) samples collected from patients with West Nile neuroinvasive disease (WNND), Veneto region, northern Italy, 2018. Graphs show the percentage of CSF samples positive for WNV IgM and WNV RNA according to the time since the onset of symptoms and the distribution of the WNV IgM and WNV RNA results in CSF collected during the first week after onset. The number of tested samples is indicated between brackets.

Laboratory diagnosis of WNND is based on the detection of WNV IgM and/or WNV RNA in CSF. During the first week after symptom onset, about 30–40% of WNND patients had negative WNV IgM testing in CSF, while WNV RNA was detectable in less than 40% of cases (Figure 7). The WNV RNA load in CSF was low (median C_T_ value, 35; range 41–31). The two tests appeared to be complementary, since most CSF samples were either WNV IgM positive or WNV RNA positive (Figure 7).

### 3.5. Phylogenetic Analysis of WNV Genome Sequences

**Figure 8:**
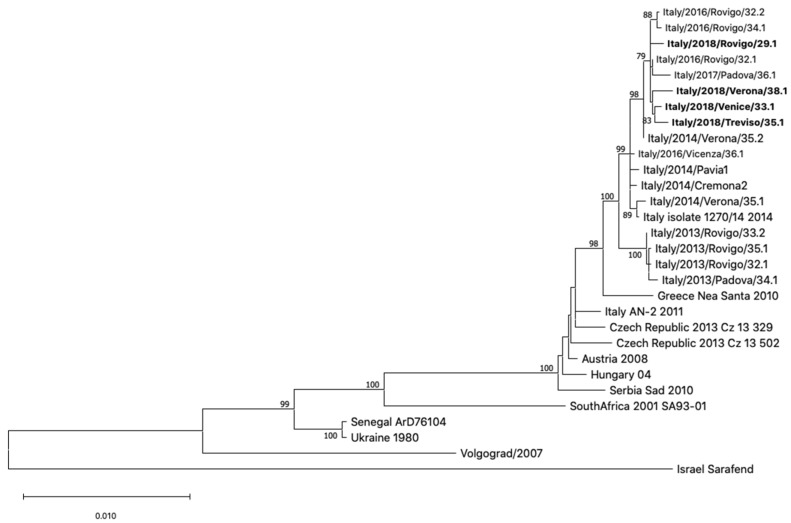
Phylogenetic tree of WNV full-genome sequences. The complete WNV genome sequences obtained in this study are highlighted in bold. The evolutionary history was inferred by using the maximum likelihood method and general time reversible model. The percentage of trees in which the associated taxa clustered together is shown next to the branches. The tree is drawn to scale, with branch lengths measured in the number of substitutions per site. This analysis involved 30 nucleotide sequences. All positions containing gaps and missing data were eliminated. There were a total of 7271 positions in the final dataset. Evolutionary analyses were conducted in MEGA X.

Real-time RT-PCR analyses and sequencing of a portion of the WNV NS5 region in samples with detectable WNV RNA showed that the virus belonged to lineage 2. WNV lineage 1 was not detected in any WNV case. Phylogenetic analysis of four WNV full genomes classified the virus within the central/southern Europe clade and showed that it was genetically very similar to WNV lineage 2 strains identified in northeastern Italy since 2013 [6,8]. In particular, the virus detected in the Veneto region in 2018 diverged from the WNV lineage 2 strain present over the years 2013–2014 (Veneto sub-clade), while it had greater similarity with the sub-clade detected in the Lombardy region since 2014, within which it constituted a distinct evolutionary group (Figure 8). The percentage of nucleotide identity among WNV genome sequences of this sub-clade was higher than 99.8%, while the percentage identity between the two sub-clades was 99.5%.

## 4. Discussion

This study, which describes a large series of 427 patients with WNV lineage 2 infection from northern Italy, allowed the epidemiological and clinical features of the currently most widespread WNV strain in Europe to be highlighted.

This study showed that the incidence of WNF progressively increased with age, while WNND incidence sharply peaked in subjects over 70 years of age. In the most affected province, the incidence of WNND and WNF were 11.6 cases and 35 cases per 100,000 population, respectively, while the incidence of WNV infection among asymptomatic blood donors was 52 cases per 100,000. As a comparison, in EU, the overall notification rate for locally acquired cases was 0.4 per 100,000 population [19]. In areas of EU countries with the highest WNV circulation, such as in Greece, Hungary, and Romania, the incidence rates ranged between 5 and 25 cases per 100,000, respectively [19].

WNV disease, especially WNND, was significantly more frequent in males than females, in agreement with epidemiological reports, which identified male gender as a risk factor for the occurrence of WNND [24]. In fact, the prevalence of anti-WNV antibodies is similar in females and males in endemic areas and the increased exposure due to outdoor activities is not sufficient to explain sex differences in the incidence of WNND [25]. Although experimental studies on the role of gender in the development of WNV encephalitis are lacking, sex differences in innate and adaptive immune responses might account for the higher prevalence and severity of WNV disease in males [26,27]. Intriguingly, in this study, the incidence rate of WNV infection was also higher in male than in female blood donors, suggesting a gender-biased risk of infection.

In patients with WNND, the case fatality rate was 22%, which was higher than the 10% reported in 2018 in the United States of America, where WNV lineage 1 is endemic [28]. Signs and symptoms of WNV infection included fever, asthenia, rash, headache, arthralgia, and myalgia, besides neurological manifestation in patients with WNND. Notably, 71% of the blood donors with WNV infection reported symptoms during follow-up visits, a proportion higher than a previous estimate of 30% in blood donors with WNV lineage 1 infection from the United States of America [29].

Laboratory diagnosis of WNV infection relies on the detection of viral RNA in blood, urine, or CSF and the demonstration of a specific antibody response in serum or CSF. Based on the results of this study and on our previous experience with the diagnosis of WNV infection [6,14,21,23], we recommend an integrated approach, combining serology and molecular testing, to increase diagnostic sensitivity and specificity. In fact, within the first week after symptom onset, WNV-specific antibodies were absent in 20–40% of cases, while WNV RNA was detectable in the blood or urine of 80% of patients. In addition, it should be kept in mind that some patients with WNND remain seronegative because of their underlying immunosuppressed condition, such as five of our patients with severe encephalitis. Moreover, subjects with previous flavivirus immunity may have a blunted or absent WNV IgM response, especially in the cases of subsequent infections with antigenically related viruses like WNV and USUV [23]. The complementarity of serology and molecular testing was observed in particular when analyzing CSF samples from patients with WNND, in which WNV RNA was generally detectable when WNV IgM antibodies were absent. The levels WNV RNA in CSF were, however, lower and detectable for a shorter time than in blood and urine, which should be considered elective biological samples for WNV molecular testing. Molecular testing provides a rapid etiological diagnosis of WNV infection, at variance with antibody detection, which needs confirmation by neutralization assays. In areas where multiple flaviviruses are endemic and co-circulate, like WNV and USUV in the Veneto region, neutralization assays against these viruses should be run in parallel [12,23]. However, in patients with previous flavivirus immunity, such as the blood donor described in this study, neutralization assays may be inconclusive.

Phylogenetic analysis of partial and full WNV genome sequences from clinical samples showed that the virus belonged to the widespread central/southern European clade of WNV lineage 2. No WNV lineage 1 was detected by either human or entomological surveillance, although we cannot exclude the presence of low-level circulation of the WNV lineage 1 in the region, unrecognized by the surveillance system. This WNV lineage 2 strain, which has been detected in the Veneto region since 2014, replaced a genetically related strain, which circulated in the same area in 2013–2014 [8,30]. A similar strain replacement was reported in the neighboring Lombardy and Emilia-Romagna regions, suggesting that this strain might have acquired improved fitness [31,32]. The anticipation of the transmission season, due to the exceptionally warm climate conditions during the 2018 spring, which increased the susceptible bird and mosquito populations and viral amplification, is considered the most important factor contributing to the dramatic increase of WNV infections in humans reported in 2018 [33]. This hypothesis was supported by the results of an epidemiological mathematical model informed by entomological data collected in the neighboring Emilia-Romagna region, northern Italy [34].

In conclusion, this study on a large series of patients with WNV infection investigated at a reference laboratory highlighted the epidemiological, clinical, and laboratory features of WNV lineage 2, which is currently spreading and causing human outbreaks in Europe, providing useful information to improve clinical and laboratory practices for WNV diagnosis.

## Figures and Tables

**Table 1:** Demographic and clinical features of subjects with confirmed West Nile virus (WNV) infection, Veneto region, 2018.

Characteristic	Clinical Condition		
	WNND (*n* = 86)	WNF (*n* = 307)	Blood Donor (*n* = 34)
Age, years			
Median (IQR)	75 (60–81) ^a^	56 (41–70) ^a^	51 (44–57)
Sex, no. (%)			
Male	56 (65) ^b^	172 (56) ^b^	27 (0.035) ^c^
Female	30 (35)	135 (44)	7 (0.017) ^c^
Clinical syndrome, no. (%)			
Encephalitis	34 (44)	0	0
Meningitis	31 (40)	0	0
Meningoencephalitis	9 (12)	0	0
Acute flaccid paralysis	2 (3)	0	0
Guillain Barré syndrome	1 (1)	0	0
Symptoms, no. (%)			
Days since onset of symptoms or index blood donation, median (IQR)	6 (1–12)	9 (1–20)	4 (2–6)
Fever	84 (97)	264 (86)	9 (26)
Median temperature, °C	39.0 (up to 41)	38.5 (up to 40.5)	38 (up to 39)
Arthralgia	20 (23)	141 (46)	7 (21)
Myalgia	21 (24)	130 (43)	12 (35)
Headache	41 (47)	138 (45)	12 (35)
Asthenia	53 (61)	184 (60)	14 (41)
Lymphadenopathy	4 (4)	34 (11)	2 (6)
Itching	0	9 (9)	4 (12)
Rash	22 (26)	138 (45)	8 (24)
Gastrointestinal symptoms	0	17 (6)	4 (12)
Asymptomatic	0	0	10 (29)
Outcome			
Death, no. (%)	19 (22)	0	0
Male	10 (53)	-	-
Female	9 (47)	-	-
Age, median years (range)	81 (58–91)	-	-

^a^ WNND vs. WNF; χ^2^ test, *p* <.0001; ^b^ Females vs Males; χ^2^ test, *p* <.005. ^c^ Percentage of WNV infected female and male blood donors out of the total number of female and male blood donors. In 2018, in the Veneto region, 66% of blood donors were males and 34% were females. – Not applicable.
